# A technical framework for costing health workforce retention schemes in remote and rural areas

**DOI:** 10.1186/1478-4491-9-8

**Published:** 2011-04-06

**Authors:** Pascal Zurn, Marko Vujicic, Christophe Lemière, Maud Juquois, Laura Stormont, Jim Campbell, Martine Rutten, Jean-Marc Braichet

**Affiliations:** 1 World Health Organization, Geneva, Switzerland; 2 World Bank, Washington DC., USA; 3 World Bank, Dakar, Senegal; 4 Instituto de Cooperación Social - Integrare, Barcelona, Spain; 5 LEI-Wageningen University, The Hague, The Netherlands

## Abstract

**Background:**

Increasing the availability of health workers in remote and rural areas through improved health workforce recruitment and retention is crucial to population health. However, information about the costs of such policy interventions often appears incomplete, fragmented or missing, despite its importance for the sound selection, planning, implementation and evaluation of these policies. This lack of a systematic approach to costing poses a serious challenge for strong health policy decisions.

**Methods:**

This paper proposes a framework for carrying out a costing analysis of interventions to increase the availability of health workers in rural and remote areas with the aim to help policy decision makers. It also underlines the importance of identifying key sources of financing and of assessing financial sustainability.

The paper reviews the evidence on costing interventions to improve health workforce recruitment and retention in remote and rural areas, provides guidance to undertake a costing evaluation of such interventions and investigates the role and importance of costing to inform the broader assessment of how to improve health workforce planning and management.

**Results:**

We show that while the debate on the effectiveness of policies and strategies to improve health workforce retention is gaining impetus and attention, there is still a significant lack of knowledge and evidence about the associated costs. To address the concerns stemming from this situation, key elements of a framework to undertake a cost analysis are proposed and discussed.

**Conclusions:**

These key elements should help policy makers gain insight into the costs of policy interventions, to clearly identify and understand their financing sources and mechanisms, and to ensure their sustainability.

## Background

Despite human resources for health having been recognized as a cornerstone to achieving better health outcomes [1], there remains a critical shortage of health workers, particularly in remote and rural areas where health outcomes tend to be significantly lower [2] and there is a considerable need for more basic health care.

Increasing the availability of health workers in remote and rural areas through improved health workforce attraction and retention is therefore crucial, not only to improve population health, but also to reach the targets set out by the health-related Millennium Development Goals [3]. Responses to increasing the availability of health workers in remote and rural areas have included a variety of initiatives at national and international level. This includes the recent launch of a WHO programme on "Increasing access to health workers in remote and rural areas through improved retention" [4].

Despite an increasing acknowledgement of the importance of health workforce retention, there is still a considerable lack of knowledge and evidence on the costs of policies intended to achieve an equitable distribution of health workers in underserved areas. Yet costing is essential for a sound selection, planning, implementation and evaluation of these policies. This lack of a systematic approach to costing represents a serious challenge for strong health policy decision making.

Indeed, while there is a growing recognition of the importance of improving access to health workers in remote and rural areas, most countries have only very limited financial resources to address this issue. This is especially true for the 57 countries identified as having a critical health workforce shortage [5]. In this context, information about the costing of policy interventions focusing on recruitment and retention in remote and rural areas contributes to making better policy decisions.

This paper proposes a framework for carrying out costing analysis of interventions to increase the availability of health workers in underserved areas in order to help policy decision makers. This paper first reviews the evidence on costing interventions to improve health workforce recruitment and retention in remote and rural areas. On the basis of this review, it provides a framework to undertake a sound costing evaluation of policy intervention to improve health workforce retention. This framework identifies key elements for a costing evaluation but also underlines the importance of identifying key sources of financing and of assessing financial sustainability. Finally, this paper discusses and investigates the role and importance of costing in a broader discussion on how to improve health workforce planning and management.

## Methodology

A literature search was conducted using a Boolean search strategy in order to judge how much literature on costing of retention strategies is readily and easily available. Our review was limited to searches in PubMed/Medline, Embase and Cochrane from 1970 to early 2010. A grey literature search was also conducted in Google Search to try and access further evidence.

The following search terms and MeSH terms and a combination thereof were used: health personnel, health care personnel, medical personnel, health professional, health care professional, health care worker, medical worker, health workforce, health care workforce, medical workforce, retention, retain, recruit, recruitment, attract, rural health services, rural, remote, medically underserved area, costs and cost analysis, cost, finance, financing, resources.

Only titles and abstracts written in English were considered. The titles and abstracts were reviewed by two reviewers based on simple inclusion/exclusion criteria. To be included, the articles had to 1) provide an indication or explanation of costs or resources involved, 2) refer to a recruitment or retention strategy for health workers, 3) have enough information in the abstract or be available in full-text from the library of the World Health Organization. Articles were excluded if they did not contain any information on costing, finance or resource use and if they were not focused on rural, remote or underserved areas.

## Results: A lack of evidence on costing of policy interventions

Literature searches have highlighted numerous studies that describe retention interventions or studies that analyse the factors that influence health workers' decisions to go to, stay in or leave rural areas, which are of great assistance in understanding why people choose to go and work in rural areas [6-9]. However, it is significantly more challenging to find evaluations of retention schemes, as shown in a recent global review where less than 50 published studies were found containing an evaluation of a retention scheme [10].

A further evidence gap confirmed by our own literature review is the lack of studies that analyze the associated implementation costs. Although many studies disclose the estimated budget for the retention strategy, very few provide any explanation or insight into how they arrived at their final budget or a clear indication of how the strategy was costed. Out of the 171 abstracts reviewed, only 9 were found to contain any relevant information related to resource use, financing or costing [11-19]. These 9 matched the inclusion criteria listed above, but even within these, the information on costing and resource use was limited. While the literature review shows that key information for a cost analysis related to health workforce retention is often limited or even absent and rarely reported in detail in descriptions or evaluations of strategies, more information is likely to be available through other sources. For example, Ministries of Health and key implementation donors might have such information. In addition, a review of the literature on how public sector and businesses use cost analysis could also provide additional relevant information.

In terms of policy-making, a lack of evidence on costs can prove to be problematic for several reasons.

Firstly, information about costs allows a better allocation of limited financial resources. For instance, in Australia, Stanley-Davies *et al. *(2005) [20] undertook a cost comparison between two approaches to improve population access to health services. They found that the cost of establishing a stand-alone service and providing outreach services in remote areas for isolated communities in north-west Queensland was about 20% costlier than transporting patients to a centralized facility.

Secondly, a cost analysis not only provides information on the feasibility and sustainability of policy interventions but also on policies regarding access to health workers by the population. In rural district hospitals in Viet Nam, Minh *et al. *(2009) [21] found that fee levels presently used were much lower than the actual costs of providing the corresponding services. This was particularly the case for surgical operations, which reflected the fact hospital services were heavily subsidized in order to allow good access for the population to these services.

Finally, costing is also a key element for sound evaluation of policy interventions [22].

One way to address such concerns is to clearly identify key elements necessary for undertaking a global costing analysis. For this, a framework for costing policy interventions is presented in the next section. This framework illustrates a global approach to costing as it also considers funding and sustainability elements.

## A framework for costing policy interventions

**Figure 1:**
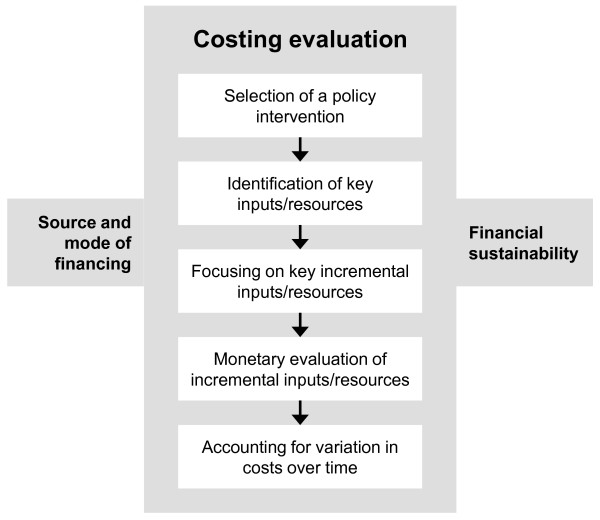
**Key elements for a costing analysis**.

In this section, key elements of a framework for costing policy interventions to increase the availability of health workers in rural and remote areas are presented and discussed. The framework depicted in Figure 1 is composed of the following three main elements, (i) costing evaluation, (ii) sources and modes of financing, and (iii) financial sustainability. This framework clearly demonstrates that all three elements are essential for a sound costing analysis.

### 1. Costing evaluation

To undertake the costing evaluation a series of steps should to be undertaken.

#### 1.1 Selection of policy intervention(s)

The first step is to clearly identify and select a single or a set of policy interventions, often referred to as a bundled intervention [23]. In the context of the WHO programme on increasing access to health workers in remote and rural areas, four main types of interventions are proposed: (i) education, (ii) regulatory interventions, (iii) financial incentives, and (iv) personal and professional support [24]. Under each category, various policies can be considered. Examples of policy interventions associated with each category are displayed in Table 1.

**Table 1:** Selected interventions to improve recruitment and retention of health workers in remote and rural areas

Category of intervention	Examples
**A. Education and continuous professional development interventions**	Building of a medical school in rural or remote area
	
	Recruitment from and training in rural areas
	
	Targeted admission of students from rural background
	
	Early and increased exposure to rural practice during undergraduate studies (diversification of location of training sites)
	
	Educational outreach programmes
	
	Community involvement in selection of students
	
	Support for continuous professional development, career paths

**B. Regulatory interventions**	Compulsory service requirements for health professionals (bonding schemes)
	
	Conditional licensing (license to practice in exchange of location in rural areas for foreign doctors)
	
	Loan repayment schemes (paid studies in exchange of services in rural areas for 4-6 years)
	
	Increased opportunities for recruitment to civil service
	
	Recognize overseas qualifications
	
	Policies enabling the production of different types of health workers (mid-level cadres, substitution, task shifting)

**C. Financial incentives (direct and indirect)**	Higher salaries for rural practice
	
	Rural allowances, including installation kit
	
	Pay for performance
	
	Different remuneration methods (fee for service, capitation etc)
	
	Loans (housing, vehicle)
	
	Grants for family education
	
	Other non-wage benefits

**D. Personal and professional support**	General improvement in rural infrastructure (housing, roads, phones, water supplies, radio communication etc
	
	Improved working and living conditions, including opportunities for child schooling and spouse employment, ensured adequate supplies of technologies and drugs
	
	Strengthening HR management support systems
	
	Supportive supervision
	
	Special awards, civic movement, and social recognition
	
	Flexible contract opportunities for part-time work
	
	Measures to reduce the feeling of isolation of health workers (professional/specialist networks, remote contact through telemedicine and telehealth)

Source: Adapted from World Health Organization, (2010) [25]

#### 1.2 Identification of key inputs/resources of the selected policy intervention

Once a policy intervention is selected, one has to identify the inputs or, in other words, the resources required to perform such a policy intervention. The perspective taken for the cost analysis should also be taken into account as it will have an impact for the identification of key inputs/resources. For example, a cost analysis from a societal perspective will not include the same inputs/resources as a cost analysis from the patient or health provider's perspective.

A comprehensive review of all inputs required could be very time consuming and arduous due to the large number of inputs which might be necessary to perform the policy intervention. Therefore, it might be appropriate to begin with the identification of the key inputs required for the intervention to inform initial planning, as well as to differentiate between capital and recurrent resources.

Examples of capital costs would usually be those related to inputs that are already in place and not under consideration to be changed (usually items with a life-span of more than one year), such as the construction of health facilities and/or purchasing of equipment. Salaries, electricity provision and allowances would be examples of current/recurrent costs [26].

The type and amount of resources required to undertake each policy intervention varies according to the characteristics of the latter. With reference to the policy interventions presented in Table 1, for instance, the building of a medical school in a rural area requires a large amount of capital resources, notably buildings and equipments. Some interventions aiming at the general improvement in rural infrastructure also call for significant amount of resources, in particular capital investments, e.g., housing, roads, water supplies, etc. However, other policy interventions like financial incentives are much less capital intensive and rely more on current financial resources like salaries, bonuses and special allowances. Other interventions like policies enabling the production of different types of health workers essentially rely on human resources such as trainers as well as education materials and equipment. Finally, some measures require very few resources like the attribution of special awards.

#### 1.3 Focusing on key incremental inputs

In order to identify the specific resources related to the policy intervention, it is important to focus on the incremental inputs, or in other words, the additional resources or inputs necessary to undertake the intervention beyond the already engaged inputs. For instance, if a country is currently scaling up its education capacity and, in addition, is also creating medical schools outside the capital city, only the additional resources required for these rural schools are to be assessed.

#### 1.4 Monetary evaluation of incremental resources

After identifying the incremental resources, their cost can be valued. Costs are normally valued in monetary units, based on prevailing prices. The objective in valuing costs is to obtain an estimate of the opportunities foregone by using the resources in the particular retention policy intervention rather than elsewhere [27].

For instance, a mid-term review of the Zambian Health Workers Retention Scheme, which aims to improve the deployment and retention of doctors in rural areas, estimated the recurrent intervention cost to be between US$621-683 per month, per contracted doctor. These incentives are significant as they represent an additional source of revenue for doctors equivalent to approximately 50% of their basic government salary [28].

Under the Zambian Health Workers Retention Scheme, a comprehensive set of interventions combining all four categories presented in Table 1, doctors serve a fixed period of three years in rural areas and in return they receive the following benefits: financial incentives, school fees, access to loans, assistance for post-graduate training and improved living conditions. By January 2005, 68 doctors had been contracted by the retention scheme [29]. Table 2 presents the main incremental cost components of the pilot experiment.

**Table 2:** Main incremental cost components

Education	
Support for postgraduate training	US$930 per contract

**Financial incentives**	

Additional rural hardship allowance	US$248-310 per month

Education allowance	US$1 676 per year, per child

Loans	US$11 160 maximum per contract

**Management, working and living environment and social support**	

Improved living conditions: funds for the maintenance of employee accommodation	US$3 104 per contracted doctor

Annual appraisal of performance and identification of training needs for capacity building	N/A

However, it is often the case that the exact amount of money required for a certain intervention may not be known. Therefore, it is pertinent to remember that calculating and gathering information on the type, amount and availability of resources required to undertake a policy intervention would also provide an insight into the eventual cost of policy intervention when information about the monetary values are missing or incomplete.

#### 1.5 Accounting for variations in costs over time

Finally, when considering costing, it is important to take the timeline into account, as the magnitude of the cost may vary significantly over time. In the Canadian province of Alberta, for example, in the context of the Rural Physician Action Plan, the number of medical students selecting approved rural teaching sites for their mandatory four week rotation in family medicine during their clinical training increased significantly between 1993 and 1997. Therefore associated costs also escalated from CAD 408 668 to CAD 1 267 154 [30]. Accounting for the timeline is also important in a context of capped funds. For instance, if a policy intervention succeeds in its objectives earlier than expected this would change the time distribution of costs and might lead to the premature finalisation of the program.

Additionally, the unit cost of key inputs may vary substantially over time. In the case of telehealth for instance, Shore *et al.*, (2007) [31] found that market changes quickly affected their cost calculations. In the course of their one-year research, which assessed the direct costs of conducting structured clinical interviews with American Indians in rural locations via telehealth, the market price of long distance communication over ISDN dropped twice and then once again after the conclusion of the study. Had the study been conducted a year later, costs would have been approximately 30% lower. Thus it is important to account for, and prepare for cost changes over time.

### 2. The source and mode of financing of the policy intervention

The second key element of the framework relates to the source and type of financing. In recent years, an increasing number of stakeholders, especially at the international level, have become more active in strengthening health systems, including the health workforce. In fact, in many circumstances, policy interventions combine different sources of funding. This diversity of actors makes it important to identify the main financiers and financing mechanisms in order to have a comprehensive understanding of the financial flows associated with the policy intervention.

Contributors include stakeholders such as international organizations or partnerships, multilateral and bilateral agencies, national public institutions such as ministries, non-governmental organizations (NGOs), private institutions, and community groups or individuals.

From an international perspective, even disease or programme specific initiatives, such as the Global Alliance for Vaccination and Immunization (GAVI), the Global Fund to Fight AIDS, Tuberculosis and Malaria and the US President's Emergency Plan for AIDS Relief (PEPFAR) have started to devote more resources to strengthening health systems, including the health workforce in recent years.

At national level, central or local authorities play a lead role, particularly the Ministry of Health. Certain policies can be financed directly from the Ministry of Health's budget (e.g. wage bonuses), while others are financed by separate government agencies (e.g. housing loan schemes financed by the Ministry of Rural Development or student loans by the Ministry of Education). This is determined by both the level of decentralization in a country and the degree of autonomy the Ministry of Health has over human resources functions. Finally, private actors and civil society, notably though local communities and NGOs, also play a role in funding. For example, in Mali, various stakeholders are also involved, as depicted here below.

Various stakeholders are directly or indirectly involved in improving doctor's distribution in rural and remote areas in Mali. Santé Sud, a French NGO, which is partially funded by the European Union and private donors, provides technical and financial support to the "Rural Doctors Association", a Malian NGO, to develop and implement strategies to attract and retain doctors in rural and remote areas. The Rural Doctors Association facilitates the installation of doctors in rural areas, notably by helping them to settle in a local community, and by providing them with specific training and some medical equipment. The Ministry of Health or local public authorities pay the doctor's wages, which are supplemented by specific benefits related to the remoteness and rurality of the practice's location. Finally, the community, notably through the "community health association", can provide additional financial resources, similar to "pay for performance" contracts.

**Figure 2:**
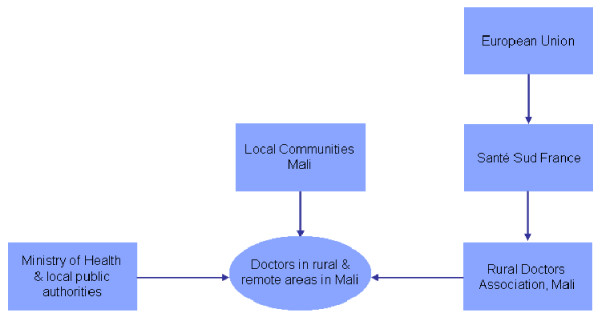
**Attracting & retaining doctors in rural areas in Mali: Main financial flows**. Source: Codjia L, Jabot F, Dubois H: Evaluation du programme d'appui à la médicalisation des aires de santé rurales au Mali, World Health Organization, Geneva, 2010 [32]

In terms of raising the financial resources for policy interventions, the latter can be financed through different avenues.

For public funding, general tax revenue is a common approach and is used in almost every country to finance certain components of health care [33]. Some taxes can be earmarked for a particular purpose. Interventions can also be financed through a deficit that is itself funded through mechanisms such as the issuing of bonds, certificates or long-term low-interest loans. Additionally, social health insurance can be a partial means to redistribute resources to improve health workforce retention in rural and remote areas. For instance, this would be possible with a reimbursement policy favouring rural health practice or with special funds dedicated for special support to rural practice. Within the private sector, either for-profit or not-for-profit funding can be accrued through private health insurance, charitable or voluntary contributions, community participation, and NGOs. More generally, out-of-pocket expenditures -- the main source of health system funding in many countries, especially in those with critical health workforce shortages -- can also be used to finance policy interventions. For example, user-fees in Uganda contributed to the funding of financial incentives for health workers in rural areas and patient utilization rates actually increased during the same period [34].

### 3. The financial sustainability of the policy interventions

Once interventions are costed and sources of financing have been identified, it is important to assess their financial sustainability. This involves judging whether financing can be secured in the medium to long term to pay for the interventions [35]. Assessing financial sustainability is important as most interventions aimed at improving rural retention require recurrent financing rather than one-off investments. If programs are not financially sustainable, there is a very high risk that they will be disrupted, which would greatly diminish effectiveness.

There is no single criterion for defining financial sustainability of interventions to improve rural retention. Rather, the central issue is to estimate program costs in the medium to long term and compare this to fiscal space and sources of financing. In making these comparisons, policy makers ought to consider several factors.

First, which agency within the government or other contributor will finance the intervention? As already discussed, there may be many government agencies involved in financing the policy. Even though the Ministry of Health is committed to a particular retention strategy, it may not be financially sustainable without the agreement of other agencies. In such cases, it is even more paramount for the Ministry of Health to demonstrate the impact of the intervention, so as to facilitate cross-government engagement and co-funding.

Second, what share of the operating budget does the retention scheme represent? In the case of financial incentives, the share of health spending devoted to remuneration varies considerably across developing countries [36]. If incentive packages are to be financed out of existing health sector budgets, then policy-makers must carefully consider whether it is feasible to reduce spending on non-remuneration items or to alter the balance between the different elements of the overall wage costs. With no well-defined benchmarks, this is challenging and must be determined on a country-by-country basis. For example, salary and allowance payments in Ghana were accounting for over 85% of recurrent health spending up until a few years ago, making it next to impossible to finance additional rural allowances [37]. In Mozambique, the statement by the Ministry of Health that home-based care volunteers should be paid 60% of the minimum wage made it difficult for some NGO's to meet this requirement on a long term basis due to budget limitation [38].

Third, how long is the budget cycle? Governments in some countries may not always plan health (and other sector) expenditures for more than one or two years ahead [39]. Similarly, while development partners are addressing the predictability of financial support, commitments to the health sector are often of a short (one to two years) duration. As a result, it is difficult to secure longer term, predictable financing for rural retention schemes. To minimize this risk, governments should adopt medium term expenditure frameworks that cover at least a two- to three-year period and budget for incentive schemes within these frameworks. In terms of donor assistance for health, longer term commitments (at least three years) are encouraged as they allow governments to raise additional revenues to absorb recurrent costs and replace donor funds at the end of the commitment period. For example, retention programs in Kenya and Malawi were partially financed through donor resources, but with commitment to a three- to six-year period, ensuring medium term sustainability [40,41]. In Malawi (see Table 3 below) DFID's long-term commitment to the Emergency Human Resources Program was evident in a 750% increase in budget support to the health sector overall whilst maintaining commitments to other specific health programming.

**Table 3:** DFID health funding to Malawi (expenditures in current prices)

	2003-04	2004-05	2005-06	2006-07	2007-08
	**(£m)**	**(£m)**	**(£m)**	**(£m)**	**(£m)**

Specific projects and programs	15.3	13.6	12.3	13.6	15.7

Budget Support allocated to Health	1.9	2.9	9.2	11	14.2

**Total**	**17.2**	**16.5**	**21.5**	**24.7**	**29.9**

Source: National Audit Office (2009) Department for International Development - Aid to Malawi.

## Discussion

From a policy perspective, it is essential to gain insight into the costs of policy interventions; therefore the framework could be of significant help to policy decision makers and could prove to be a major determinant of the success of policy interventions. In particular, this framework also emphasizes the importance of clearly identifying and understanding the financing sources and mechanisms related to the policy interventions, as well as assessing their sustainability.

While such a framework brings key elements for a sound costing of health workforce retention schemes to the forefront, it appears that some specific issues remain complex and deserve further attention or research. Firstly, combining information both on incremental cost and outcomes of policy interventions are instrumental to the selection of the most appropriate intervention. Such an approach would allow the undertaking of more global cost analyses such as cost-effectiveness, cost-benefit or cost-utility analyses. In practice, identifying the incremental costs and outcomes may not always be an easy task. Nonetheless, they must be carefully measured as otherwise serious biases may be portrayed in the reported results of the intervention.

Secondly, as success in terms of retention is associated with length/duration of practice, accounting for the time-span of both effectiveness and costs is important. The inclusion of time-to-event objectives (i.e., number of retained health worker after two years, after four years, etc.) and time-bound cost indicators (i.e., monthly or yearly costs) should be encouraged, as they contribute to better monitoring and understanding of cost evolution over several years. This in turn facilitates the development of policies that integrate this continuum.

Thirdly, a cost analysis should also be an integral part of human resources for health planning development. Indeed, planning not only involves determining the future human resources for health requirements of a population, but entails developing training capacity and the appropriate incentive packages that will produce and retain the required health care workforce. Cost analysis is therefore essential to help address these health labour market complexities and specificities in order to achieve an adequate supply and demand of health personnel.

Fourthly, the dissemination of guidance and evidence about cost analysis is essential in order to address the lack of information and knowledge on how to cost interventions. Dissemination would help inform and reinforce the debate on policies to improve attraction and retention in rural and remote areas. Cross-country cost comparisons of similar policy interventions, notably through the use of standardized costing tools, would surely provide interesting and useful insights for policy makers and contribute to global efforts towards health systems strengthening.

Finally, while costs may often appear too high and deter some policy makers, having a cost analysis leads to a more comprehensive and informed perspective through identifying the resources involved, the sources of financing and their sustainability. If policy makers combine these elements with an evaluation of the impact of the policy intervention, this may indeed lead to the selection of costly interventions, but they will be well funded, sustainable and effective.

## Conclusions

Gaining insight into the costs of policy interventions is key to ensure successful policy interventions. The proposed framework facilitates and encourages the systematic costing of health workforce retention schemes. Central to this framework are the series of steps to undertake a costing evaluation, including the identification and selection of key elements, their monetary valuation, and accounting for the variation of costs over time. Also central to this framework is the identification and understanding of financing sources and mechanisms related to the policy interventions, as well as ensuring their sustainability.

## Competing interests

The authors declare that they have no competing interests.

## Authors' contributions

PZ designed and conceptualized the study. PZ, MV, CL, MJ, LS, JC, MR and JMB provided inputs for the draft. PZ and LS revised and finalized the draft. All authors read the final draft and approved it for submission.
